# Anterior cruciate ligament primary repair revision rates are increased in skeletally mature patients under the age of 21 compared to reconstruction, while adults (>21 years) show no significant difference: A systematic review and meta‐analysis

**DOI:** 10.1002/ksa.12239

**Published:** 2024-07-05

**Authors:** Sebastian Rilk, Gabriel C. Goodhart, Jelle P. van der List, Fidelius Von Rehlingen‐Prinz, Harmen D. Vermeijden, Robert O'Brien, Gregory S. DiFelice

**Affiliations:** ^1^ Department of Orthopaedic Surgery, Hospital for Special Surgery, New York‐Presbyterian Weill Medical College of Cornell University New York New York USA; ^2^ Medical University of Vienna Vienna Austria; ^3^ Department of Orthopaedic Surgery, Atrium Health Wake Forest Baptist Wake Forest University School of Medicine Winston‐Salem North Carolina USA; ^4^ Department of Trauma and Orthopaedic Surgery University Medical Center Hamburg‐Eppendorf Hamburg Germany; ^5^ Department of Orthopaedic Surgery, Amsterdam UMC University of Amsterdam Amsterdam The Netherlands; ^6^ Boston University Chobanian & Avedisian School of Medicine Boston Massachusetts USA

**Keywords:** ACL preservation, ACL primary repair, bridge‐enhanced ACL restoration, dynamic intraligamentary stabilization

## Abstract

**Purpose:**

To evaluate the impact of age as a risk factor on the revision rates of anterior cruciate ligament (ACL) primary repair (ACLPR), dynamic intraligamentary stabilization (DIS) and bridge‐enhanced ACL restoration (BEAR) compared to ACL reconstruction (ACLR).

**Methods:**

A systematic literature search was performed for comparative studies comparing outcomes for ACLPR, DIS or BEAR to ACLR. A random‐effects meta‐analysis was performed to assess nondifferentiated and age‐differentiated (skeletally mature patients ≤21 and >21 years) ACL revision and reoperation risk, as well as results for subjective outcomes. Methodological study quality was assessed using the Risk of Bias Tool 2.0c and Methodological Index for Nonrandomized Studies tools.

**Results:**

A total of 12 studies (*n* = 1277) were included. ACLR demonstrated a lower nonage‐stratified revision risk at 2 years versus ACLPR, DIS and BEAR, but a similar revision risk at 5 years when compared to DIS. However, an age‐stratified analysis demonstrated a significantly increased ACLPR revision risk as compared to ACLR in skeletally mature patients ≤21 years of age (risk ratios [RR], 6.33; 95% confidence interval [CI], 1.18–33.87, *p* = 0.03), while adults (>21 years) showed no significant difference between groups (RR, 1.48; 95% CI, 0.25–8.91, n.s.). Furthermore, DIS reoperation rates were significantly higher than respective ACLR rates (RR, 2.22; 95% CI, 1.35–3.65, *p* = 0.002), whereas BEAR (RR, 1.07; 95% CI, 0.41–2.75, n.s.) and ACLPR (RR, 0.81; 95% CI, 0.21–3.09, n.s.) showed no differences. IKDC scores were equivalent for all techniques. However, ACLPR exhibited significantly better FJS (mean difference, 11.93; 95% CI, 6.36–17.51, *p* < 0.0001) and Knee injury and Osteoarthritis Outcome Score Symptoms (mean difference, 3.01; 95% CI, 0.42–5.60, *p* = 0.02), along with a lower Tegner activity reduction.

**Conclusions:**

ACLPR in skeletally mature patients ≤21 years of age is associated with up to a six‐fold risk increase for ACL revision surgery compared to ACLR; however, adults (>21 years) present no significant difference. Based on the current data, age emerges as a crucial risk factor and should be considered when deciding on the appropriate treatment option in proximal ACL tears.

**Level of Evidence:**

Level III.

**Abbreviations**
ACLPR: ACL primary repair
ACLR: ACL reconstruction
ATT SSD: anterior‐tibial translational side‐to‐side difference
BEAR: bridge‐enhanced ACL restoration
BPTB: bone‐patellar tendon‐bone
DIS: dynamic intraligamentary stabilization
FJS: Forgotten Joint Score‐12
HT: hamstring tendon
IKDC: International Knee Documentation Committee
IQR: interquartile range
KOOS: Knee injury and Osteoarthritis Outcome Score
LET: lateral extra‐articular tenodesis
LOE: level of evidence
MINORS: Methodological Index for Nonrandomized Studies
MOON: Multicenter Orthopaedic Outcomes Network
PASS: patient acceptable symptom state
QT: quadricep tendon
RCT: randomized controlled trial
RoB 2: Risk of Bias Tool 2.0c
ROH: removal of hardware
RR: risk ratio
SD: standard deviation
VAS: Visual Analogue Sale

## INTRODUCTION

The resurgence of interest to preserve and repair the anterior cruciate ligament (ACL) [40, 55, 60] has led to an increasing number of comparative studies at short‐ [6, 11, 17, 18, 25, 39, 44, 45, 64, 78] and mid‐term follow‐up [22, 27, 31], as well as a high number of systematic reviews and/or meta‐analyses [26, 32, 36, 49, 53, 54, 58, 65, 73, 74, 76, 82]. Even though contemporary techniques, such as ACL primary repair (ACLPR) [23, 51, 59, 70], (ii) dynamic intraligamentary stabilization (DIS) [13] and (iii) the bridge‐enhanced restoration technique (BEAR) [19, 46], share the common objective of a preservation‐first approach [72], they differ in terms of surgical indications, techniques and rehabilitative methods [13, 23, 46, 51, 59]. Previous reviews, however, used the term ‘ACL repair’ in a historical context and overlooked technique‐defining characteristics, as they pooled outcomes for ACLPR, DIS and BEAR [49, 53, 58]. This has introduced heterogeneity into conclusions and opportunities were missed to investigate approach‐specific risk factors [49, 53, 58].

To avoid having history repeat itself, with insufficient outcomes at mid‐ to long‐term follow‐up [15, 37, 66], contemporary repair approaches must focus on refined patient selection criteria based on technique‐specific risk factors. With this in mind, Vermeijden et al. presented the age of 21 as an influential age cut‐off regarding the risk of reinjury, emphasising the importance of age stratification [78]. Subsequently, ACLPR and BEAR studies have presented younger age as a risk factor for ACL revision, with high rates of ACL revision in patients ≤21 years of age and low rates in patients aged >21 [17, 63, 76]. As of yet, however, no review has compared ACLPR, DIS and BEAR revision rates with regard to age [36, 49, 53, 58].

Therefore, the purpose of this study was to perform a systematic review and meta‐analysis to evaluate the impact of age as a risk factor on the revision rates of ACLPR, DIS and BEAR compared to ACLR. This should provide clinicians with extended knowledge regarding the risk factor age, helping to further refine the indication spectrum for ACLPR and to guide the treatment decision‐making for proximal ACL tears. It was hypothesised that skeletally mature patients ≤21 years of age would be associated with an increased risk for subsequent ACL injury and the need for ACL revision surgery.

## MATERIALS AND METHODS

### Search strategy

This systematic review was conducted according to the guidelines of the Cochrane Handbook [7] and followed the Preferred Reporting Items for Systematic Reviews and Meta‐Analyses (PRISMA) guidelines using the PRISMA checklist [43]. Additionally, the review was registered with PROSPERO (ID CRD42023468060). A systematic literature search was conducted independently by two authors (S. R. and G. C. G.) in July 2023 of English articles in PubMed, Embase and the Cochrane Library. The electronic database search employed the following search terms and combinations: (‘ACL’ OR ‘anterior cruciate ligament’) AND (‘repair’ OR ‘reinsertion’ OR ‘reattachment’ OR ‘healing’ OR ‘suture augmentation’ OR ‘dynamic intraligamentary stabilisation’ OR ‘bridge‐enhanced restoration’ OR ‘bridge‐enhanced’ OR ‘restoration’). Additionally, reference lists of all included studies were reviewed to identify articles that were potentially missed in the systematic search. After removing duplicates, the same two authors (S. R. and G. C. G.) independently reviewed titles and abstracts, applying predefined inclusion and exclusion criteria. Any discrepancies in literature selection were resolved through consensus.

### Eligibility and exclusion criteria

The review considered adults (>21 years) and skeletally mature patients ≤21 years of age with ACL tears treated with ACLPR, DIS or BEAR, comparing them to patients undergoing ACL reconstruction (ACLR) to evaluate outcomes based on ACL revision rates, patient‐reported outcomes measurements (PROMs) and/or instrumented laxity measurements at 2‐ and/or 5‐year follow‐up. Studies were limited to comparative clinical studies with a level of evidence (LOE) 1–3 reported in English full text. Further exclusion criteria included patients with multiligamentous knee injuries. In cases where two studies reported on the same cohort [22, 25, 27, 29, 39], outcomes were not duplicated and data were only presented for the study with the higher LOE and/or longer follow‐up period [22, 27, 29].

### Data extraction

Data from the included studies were independently extracted by two authors (S. R. and G. C. G.) based on predetermined key items, which encompassed (i) general study design information, (ii) patient demographics, (iii) details of surgical and rehabilitative procedures, (iv) records of adverse events, (v) clinical outcomes (instrumented laxity measurements) and (vi) PROMs. In cases of initial disagreement during this process, consensus was reached between the same two authors. Data were directly extracted from the articles, and attempts were made to contact all 12 authors [1, 4, 11, 17, 18, 22, 27, 31, 38, 45, 47, 77] via e‐mail to gain further insight into the raw data set if necessary. Of these authors, eight [1, 11, 17, 18, 27, 31, 38, 77] provided additional data from the raw data set, which had not been reported in their respective original publications. Missing basic demographic parameters were added, and six authors [11, 17, 18, 27, 31, 38] were able to provide additional information on the type of reoperation, the reinjury time, the reinjury mechanism and/or the age at ACL revision, allowing for a more granular analysis, with a specific focus on the age‐differentiated ACL revision analysis according to predefined age groups: skeletally mature patients ≤21 years of age and adults (>21 years).

### Primary and secondary outcome measures

The primary outcome parameter of this meta‐analysis was the number of patients reported undergoing ACL revision surgery, with an additional age‐stratified subgroup analysis. Secondary parameters analysed were reoperation rates (subsequent need for surgery on ipsilateral knee other than ACL revision) and PROMs International Knee Documentation Committee (IKDC) subjective score [33], Knee injury and Osteoarthritis Outcome Score (KOOS) [8], Lysholm Score [5], pre‐ and postoperative Tegner Score [71], Pain Scores (Visual Analogue Scale [VAS]) [57] and Forgotten Joint Score‐12 (FJS) [3]. Additional outcomes were assessed qualitatively and in a tabulated fashion to avoid inappropriate pooling and heterogeneity: time of ACL reinjury (months after primary surgery), type of ACL reinjury mechanism (traumatic or atraumatic), instrumented anterior‐tibial translational side‐to‐side difference (ATT SSD).

To further interpret PROMs, presented outcomes for IKDC subjective scores, KOOS, Lysholm and FJS were compared to recently defined patient acceptable symptom state thresholds for ACLPR: Subjective IKDC 73.6, Lysholm 89.0, KOOS Pain 91.7, KOOS Symptoms 85.7, KOOS Activities of Daily Living 99.0, KOOS Sport/Recreation 75.0, KOOS Quality of Life 62.5 and FJS 68.8 [17].

### Risk of bias assessment

The methodological quality of all included studies was assessed by two independent investigators (S. R. and F. R. P.). The inter‐rater reliability was measured using Cohen's *κ* coefficient [42] and a *κ* value of 0.75 or greater was considered of excellent agreement [16]. For randomised controlled trials (RCTs), the Cochrane Risk of Bias Tool 2.0 (RoB 2) [69] was applied, while nonrandomized studies were assessed using the Methodological Index for Nonrandomized Studies (MINORS) [67]. The RoB 2 evaluates RCTs across five domains of bias, and according to an algorithm‐based risk of bias judgement, studies are categorised as ‘Low’ or ‘High’ risk of bias or with ‘Some concerns’. The MINORS tool comprises 12 fundamental questions for nonrandomized trials each of which could be scored with 0 points for ‘not reported’, 1 point if ‘reported but inadequate’ or 2 points if ‘reported and adequate’. The inter‐rater reliability was calculated as excellent for both the MINORS and Rob 2 assessments, with 0.8 and 0.9, respectively. Any discrepancies were resolved through discussion. A LOE assessment was carried out following the Oxford Centre for Evidence‐Based Medicine's ‘Levels of Evidence’ [34]. In addition, Funnel plots and the Egger test were employed to assess publication bias [12].

### Statistical analysis

The outcomes for this review are presented in tables as mean ± standard deviation (SD) for numerical outcome variables, absolute values and percentages for dichotomous outcomes. When publications exclusively reported median or interquartile range [27, 38, 45], mean and SD were estimated as previously described [24, 79]. RevMan version 5.3 (The Cochrane Collaboration) was used to perform a random‐effects meta‐analysis of mean differences for continuous outcome variables (PROMs) and a relative risk random‐effects meta‐analysis for dichotomous variables (revision and reoperation rates). Risk ratios (RR) with estimates of uncertainty were calculated and presented using forest plots. This accounted for all studies with two or more references available per subgroup, reporting on the primary or secondary outcome parameters of this analysis. To assess and characterise the heterogeneity among eligible studies, the Cochran *Q* statistics and *I* ^2^ statistics were applied. Heterogeneity was considered low if the estimated *I* ^2^ values were under 40% or high if greater than 75% [7]. Data not included in the relative risk random‐effects meta‐analysis are presented in a tabulated and qualitative in‐text fashion. Statistical analysis was performed using SPSS Statistics version 28 (IBM Corporation). Statistical significance was set at a *p* value of <0.05.

## RESULTS

### Study characteristics

**Figure 1:**
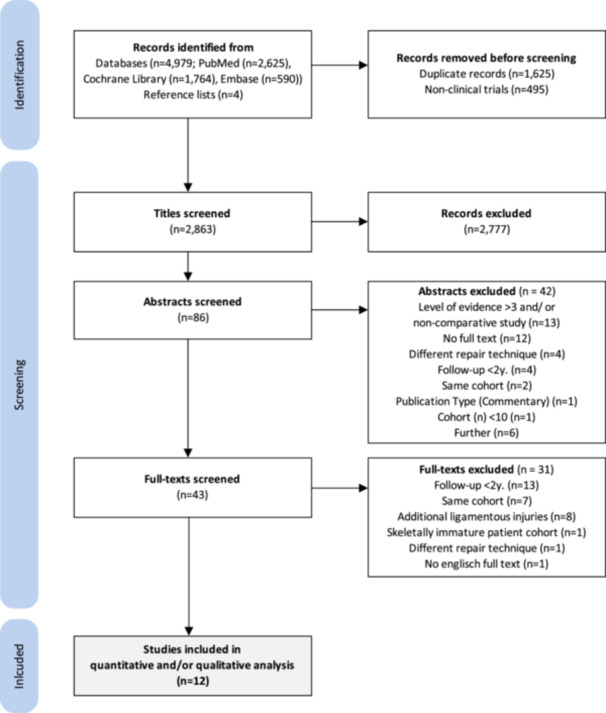
Flow diagram depicting the study selection process.

**Figure 2:**
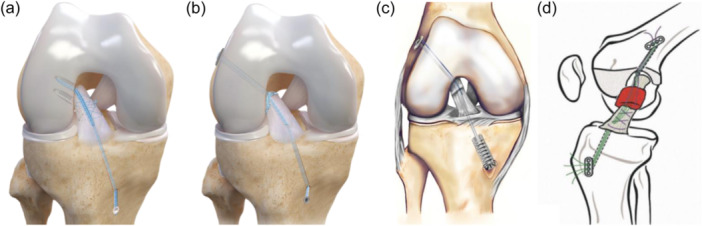
Modern day anterior cruciate ligament (ACL) repair techniques: (a) ACL primary repair with dual suture anchor fixation [59] (Arthrex), (b) ACL primary repair with cortical button fixation (Arthrex), (c) dynamic intraligamentary stabilisation (reproduced from Ateschrang et al. [2] with permission) and (d) bridge‐enhanced ACL restoration (reproduced from Murray et al. [45] with permission).

A total of 4983 studies were yielded in the initial literature search (Figure 1 *)*. After this search, a total of 12 comparative studies with 1227 patients, which, respectively, compared ACLPR [1, 11, 17, 18, 31, 77] (*n* = 364), DIS [4, 22, 27, 38] (*n* = 126) or BEAR [45, 47] (*n* = 75) against ACLR (*n* = 662), were included in the final qualitative synthesis and the risk of bias assessment (Figure 2). Mean follow‐up ranged from 2.0 to 6.1 years. Tables 1, 2, 3, 4 display all demographic, clinical and methodological characteristics of the included studies.

**Table 1:** Patient demographics and preoperative clinical characteristics.

	Number of patients			Follow‐up length, year			Age, year			Female, *n* (%)			Delay injury surgery, week			Tegner pre		
References	Total	ACLPR	ACLR	ACLPR	ACLR	Sign.	ACLPR	ACLR	Sign.	ACLPR	ACLR	Sign.	ACLPR	ACLR	Sign.	ACLPR	ACLR	Sign.
ACL primary repair																		
FU ≥ 2 years																		
Ferreira et al. [17]	150	75	75	2.5 ± 0.5	2.5 ± 0.4		40 ± 11 (16–62)	38 ± 11 (19–71)		46 (61)	48 (64)		8.8 ± 11.6 (0.8–36.4)	9.2 ± 5.6		6.1 ± 1.2 (2–9)	6.1 ± 1.8 (3–9)	
Ferretti et al. [18]	100	53	47	2.1 ± 1.8 (2.0–2.6)			33 ± 12 (18–57)	25 ± 11 (18–47)	0.19	21 (40)	17 (36)	0.80	1.3 ± 0.4	1.3 ± 0.4	0.84	7^§^	7^§^	0.70
Achtnich et al. [1]	41	20	21	2.3 ± 0.6 (2.0–2.6)			30 ± 9	34 ± 4	0.371	8 (40)								
Douoguih et al. [11]	60	30	30	≥2	≥2		28 ± 5 (18–37)	26 ± 4 (17–33)	0.56	17 (57)	16 (53)					3.9 ± 2.9	2.9 ± 3.3	0.07
Vermeijden et al. [77]	83	49	34	2.5 ± 0.8 (1.6–4.8)	3.0 ± 1.1 (1.7–4.8)	0.012	34 ± 11 (16–55)	29 ± 11 (16–52)	0.047	25 (49)	14 (41)		46.3 ± 52.7	98.0 ± 117.6	0.202			
FU ≥ 5 years																		
Hopper et al. [31]	410	137	273	5.3 ± 1.5 (2.5–8.8)	5.4 ± 1.8 (1.8–8.0)		35 ± 14 (13–60)	28 ± 9 (15–57)		60 (44)	49 (18)		5.1 ± 4.4 (0.4–20.0)	104.6 ± 160.4 (0–1440)		6.8 ± 1.5	6.5 ± 1.6	

	Total	DIS	ACLR	DIS	ACLR	Sign.	DIS	ACLR	Sign.	DIS	ACLR	Sign.	DIS	ACLR	Sign.	DIS	ACLR	Sign.
Dynamic intraligamentary stabilisation																		
FU ≥ 2 years																		
Kayaalp et al. [38]	45	15	30	4.2 ± 1.1 (2.2–5.4)	4.0 ± 1.0 (2.2–5.6)	NS	28 ± 10 (16–47)	27 ± 10 (17–49)	NS	3 (20)	6 (20)	NS	2.2 ± 2.0	7.1 ± 2.8	<0.001	5.7 ± 4.9		
Bieri et al. [4]	106	53	53	≥2	≥2		30 ± 9 (18‐55)	31 ± 8	0.40	10 (19)	10 (19)	1.00	2.0 ± 1.8	7.1 ± 3.9	<0.001			
FU ≥ 5 years																		
Hoogeslag et al. [27]	48	24	24	5.2 ± 0.3 (5.0–6.3)			19 ± 13	22 ± 4 (19‐25)	0.693	5 (21)	6 (25)	0.731	3.4 ± 0.8	13.3 ± 5.7		8.0 ± 1.6	8.5 ± 1.6	0.893
Glasbrenner et al. [22]	64	34	30	6.1 ± 0.3 (4.7–6.1)	5.3 ± 0.2 (4.8–5.8)		28 ± 12 (18–50)	27 ± 12 (18–50)		15 (44)	8 (27)		2.0 ± 0.7	2.2 ± 1.0		5.6 ± 1.2		

	Total	BEAR	ACLR	BEAR	ACLR	Sign.	BEAR	ACLR	Sign.	BEAR	ACLR	Sign.	BEAR	ACLR	Sign.	BEAR	ACLR	Sign.
Bridge‐enhanced ACL restoration																		
FU ≥ 2 years																		
Murray et al. [45]	100	65	35	≥2	≥2		18 ± 3 (13–35)	18 + ± 6 (13–35)	0.76	37 (57)	19 (54)	0.84	5.1 ± 1.4	5.5 ± 1.1	0.15			
Murray et al. [47]	20	10	10	≥2	≥2		24 ± 5 (18–25)	25 ± 6 (19–34)	NS	6 (60)	8 (80)	NS	3.0 ± 0.7 (1.60–4.0)	7.6 ± 2.4 (3.40–11.4)	<0.001			

*Note*: Data presented as mean ± SD (range), if not indicated otherwise: ^§^median.

Abbreviations: ACL, anterior cruciate ligament; ACLPR, ACL primary repair; ACLR, ACL reconstruction; BEAR, bridge‐enhanced ACL restoration; BMI, body mass index; DIS, dynamic intraligamentary stabilisation; FU, follow‐up; NS, nonsignificant.

**Table 2:** Study methodology characteristics.

References	Study design (LOE)	PRO/RP	Matching		Recruitement period	Inclusion criteria	Exclusion criteria	Lost to FU, *n* (%)	Failure definition
ACL primary repair									
FU ≥ 2 years									
Ferreira et al. [17]	Cohort study (3)	RP	Yes	Propensity score matching (age, time between injury and surgery, sex, BMI, SSD ap laxity, meniscal lesion, Tegner, participation in pivoting or contact sports	09/2017–05/2019	ACL tear <12 months injury to surgery	Skeletally immature, history of ipsilateral knee surgery, major concomitant procedure (MLIK, LET, osteotomy)	2 ACLPR (3), 5 ACLR (6)	Revision ACL surgery after graft rupture
Ferretti et al. [18]	Cohort study (2)	PRO	No		01/2019–06/2020	Complete tear of the ACL (clinical and MRI confirmed) with reduceable tissue	Surgery >15 days of injury, age <18 years, history of IL knee surgery, additional knee ligament injury (except for ALL), Kellgren–Lawrence grade 3/4, professional athletes, refusal to participate	0 (0)	Failure of the repaired ACL, or graft rupture of the reconstructed ACL
Achtnich et al. [1]	Case–control study (3)	RP	No		01/2010–12/2013	Acute (≤6 weeks) proximal ACL tear (MRI confirmed), concomitant meniscus lesions	Previous knee ligament surgery, additional IL or CL ligament injuries	0 (0)	ACL reinjury, recurrent instability
Douoguih et al. [11]	Cohort Study (2)	PRO	No		03/2018–01/2020	ACL type 1/2 tear (MRI confirmed), age ≥14 years	Previous IL or CL ACL surgery, concomitant IL ligamentous knee injury or pre‐existing IL osteoarthritis	3 (5)	ACL reinjury
Vermeijden et al. [77]	Cohort study (3)	RP	No		05/2012–05/2012	ACLPR: proximal ACL tears (i.e., distal remnant length sufficient to reattach ligament back to the femoral footprint) and sufficient tissue quality to withhold suture passing; ACLR: nonrepairable tears (i.e., mid‐substance tear or insufficient tissue quality to withhold sutures)	Multiligmanetous injured knees, pre‐existing osteoarthritis, skeletally immaturity, failure	56 (40)	
FU ≥ 5 years									
Hopper et al. [31]	Case–control study (3)	RP	No		2011‐2018	Repair: acute proximal tears with adequate ACL tissue quality within 3 months of injury	Multiligament injuries or surgery to the ALL ligament	3 ACLPR (2), 1 ACLR (0.4)	Ligament/graft rerupture
Dynamic intraligamentary stabilisation									
FU ≥ 2 years									
Kayaalp et al. [38]	Case–control study (3)	RP	Yes	1:2 matching (age, sex, Tegner, concomitant injuries)	03/2015–09/2018	General: Acute ACL injury (max 4 weeks), DIS: proximal or middle third ACL tear	Concomitant ligamentous or meniscal injuries necessitating repair	1 DIS (6), 2 ACLR (6)	Ligament/graft rerupture, SSD ATT > 3 mm, or subjective instability
Bieri et al. [4]	Case–control study (3)	RP	Yes	*n*:1 propensity score matching (age, sex, working category, time between rupture and surgery)	2011–2012	General: covered by Suva compulsory accident insurance, primary traumatic ACL tear, age 18–55; ACLR: autograft, injury‐surgery ≥360 days, no initial conservative treatment	incomplete patient records (*n* = 9), conservative treatment approach first (*n* = 8), re‐rupture of the ACL during FU (*n* = 7), concomitant knee injuries (*n* = 35)	2 years: 21 (15), 5 years: 21 (25)	ACL revision surgery, due to traumatic reinjury or chronic instability
FU ≥ 5 years									
Hoogeslag et al. [27]	RCT (1)	PRO	RCT		01/2015–03/2016	Age 18–30, diagnosed ACL rupture (history, exam, MRI), surgery within 21 days, TAS 5–10	Concomitant ligamentous lesions, history of CL/IL knee surgery, meniscal lesions needing surgical repair, full‐thickness cartilage lesions, osteoarthritis	1 (4) DIS, 3 (11) ACLR	Findings at physical examination, subjective instability and/or graft rupture
Glasbrenner et al. [22]	RCT (1)	PRO	RCT		2014–2015	Age 18–50, acute proximal or mid‐substance ACL tear (positive Lachman or pivot‐shift test), surgery within 3 weeks after injury, stable meniscal lesions	Previous IL/CL knee injuries, concomitant meniscal, cartilage and collateral ligament injuries altering operative procedure or rehabilitation programme	2 (2)	Patient undergoing revision ACL reconstruction
Bridge‐enhanced ACL restoration									
FU ≥ 2 years									
Murray et al. [45]	RCT (1)	PRO	RCT		05/2016–06/2017	Complete ACL tear, 13 to 35 years, <45 days from injury, closed physes, at least 50% of the length of the ACL attached to the tibia	General: history of IL knee surgery, previous knee infection, nicotine/tobacco use, past 6‐month corticosteroid use, chemotherapy, diabetes, inflammatory arthritis), displaced medial meniscus bucket‐handle tear that required repair (all other types included), full‐thickness chondral injury, grade 3 MCL injury, patellar dislocation, operative posterolateral corner injury. BEAR: <50% ACL length attached to the tibial footprint	1 (2)	Incidences of ACL failure requiring a second ipsilateral ACL procedure
Murray et al. [47]	Cohort study (2)	PRO	No		02/2015–10/2015	Complete ACL tear, 13 to 35 years, BEAR: <1 month from injury, ACLR: <3 months from injury, at least 50% of the length of the ACL attached to the tibia		0 (0)	Absence of intact continuous fibres in the expected region of the repair or graft

Abbreviations: ACL, anterior cruciate ligament; ACLPR, ACL primary repair; ACLR, ACL reconstruction; ALL, antero‐lateral ligament; BEAR, bridge‐enhanced ACL restoration; BMI, body mass index; CL, contra‐lateral leg; DIS, dynamic intraligamentary stabilisation; ext., extension; flex., flexion; FU, follow‐up; IL, ipsi‐lateral leg; LET, lateral extra‐articular tenodesis; LOE, level of evidence; MCL, medial collateral ligament; PRO, prospective; RCT, randomised controlled trial; RP, retrospective; SSD ATT, side‐to‐side difference anterior‐tibial‐translation.

**Table 3:** Surgical ACL technique and rehabilitative regimen.

	ACLPR/DIS/BEAR technique								ACLR technique	Additional procedure	Single surgeon	Rehabilitation	
References	Arthroscopic, yes/no	Femoral fixation	Fixation angle	SA, yes/no	SA fixation angle	Repair criteria	Tear location,^a^ %	Tear type classification	Graft		Yes/no (*n*)	Weight bearing	Immobilisation
ACL primary repair													
FU ≥ 2 years													
Ferreira et al. [17]	Yes	CBF	90°	Yes	Full extension	Proximal ACL tear, good tissue quality (normal macroscopic appearance without fraying or splitting), reducibility of the remnant (4 CROSS test)	100/−/−	Sherman Type I + II	BPTP (17), HT (83)		No (2)	Immediatetly	Full ROM
Ferretti et al. [18]	Yes	CBF		No		Proximal and reduceable ACL tear	100/−/−	Sherman Type I + II	HT (100)	LET: Coker‐Arnold (Modified MacIntosh)	Yes	As tolerated	full ext. Week 1, 0–90° Weeks 2–4
Achtnich et al. [1]	Yes	sSAF		No		Proximal avulsion tear and sufficient tissue quality	100/−/−	Sherman Type I + II	HT (100)			Partial 6 weeks	full ext. Week 0–2, 0–90° Week 3–6
Douoguih et al. [11]	Yes	CBF		Yes	Full extension	Proximal ACL tear (MRI confirmed)	100/−/−	Sherman Type I + II	QT (57), BPTP (43)		Yes	As tolerated	
Vermeijden et al. [77]	Yes	dSAF	90/115°	Yes^b^	Full extension	Proximal ACL tears (i.e., distal remnant length sufficient to reattach ligament back to the femoral footprint) and sufficient tissue quality to withhold suture passing	100/−/−	van der List Type I + II	Allograft/hybrid (50), BPTP (26), HT (24)		Yes	Immediately	Brace 4 weeks
FU ≥ 5 years													
Hopper et al. [31]	Yes	CBF		Yes		Acute proximal tears (≤3 months), adequate tissue quality	100/−/−		HT (100)		No (2)		
Dynamic Intraligamentary stabilisation													
FU ≥ 2 years													
Kayaalp et al. [38]	Yes	CBF		No		Acute (≤4 weeks) proximal or mid‐substance ACL tears			HT (100)		Yes	Immediately	DIS: Brace locked in ext. 5 days; ACLR: no restriction
Bieri et al. [4]	Yes	CBF		No					HT (67), BPTP (27), QT (6)		No		
FU ≥ 5 years													
Hoogeslag et al. [27]	Yes	CBF	0°	No		Randomised	83/13/4		HT (100)		Yes		DIS: Long leg splint locked in ext. first 5 days; ACLR: no restriction
Glasbrenner et al. [22]	Yes	CBF	Close to full ext.	No		Randomised, acute (≤3 weeks) proximal or mid‐substance ACL tears	91/9/−		HT (100)		No (4)	first 2 weeks 20 kg, then full	Brace
Bridge‐enhanced ACL restoration													
FU ≥ 2 years													
Murray et al. [45]	50 mm arthrotomy	CBF		Yes		Randomised, acute (<45 days) complete proximal and mid‐substance ACL tears, closed physes	−/100/−	MRI: 50% ACL length attached to the tibia	HT (94), BPTB (6)		No (3)	Partial 2 weeks, 2–4 weeks as tolerated with crutches	0–50° Week 0‐2, 0–90° Week 3–6
Murray et al. [47]	50 mm arthrotomy	CBF		Yes		Acute (<1 month) complete proximal and mid‐substance ACL tears	−/100/−		HT (100)		Yes		

Abbreviations: ACL, anterior cruciate ligament; ACLPR, ACL primary repair; ACLR, ACL reconstruction; BEAR, bridge‐enhanced ACL restoration; BPTB, bone‐tendon tendon‐bone autograft; CBF, cortical button fixation; DIS, dynamic intraligamentary stabilisation; dSAF, dual suture anchor fixation; ext., extension; flex., flexion; FU, follow‐up; HT, hamstring tendon autograft; LET, lateral extra‐articular tenodesis; QT, quadricep tendon autograft; SA, suture augmentation; SAF, suture anchor fixation; sSAF, single suture anchor fixation.

^a^ Tear location: proximal/mid‐substance/distal

b Patients with a greater risk of failure, an internal suture augmentation was added to the repair;

**Table 4:** Concomitant injuries.

	Meniscal treatments, total		Menisectomy						Meniscal Repair						MCL		ALL	
			Total	Med.		Total			Total	Med.		Total						
References	ACLPR	ACLR	ACLPR		Lat.	ACLR	Med.	Lat.	ACLPR		Lat.	ACLR	Med.	Lat.	ACLPR	ACLR	ACLPR	ACLR
ACL primary repair																		
FU ≥ 2 years																		
Ferreira et al. [17]	14 (19)	0 (0)	3	1	2	3	2	1	11	6	5	15	7	8				
Ferretti et al. [18]	13 (25)	12 (21)															53 (100)	47 (100)
Achtnich et al. [1]	15 (75)	14 (67)	0			0			0			0						
Douoguih et al. [11]																		
Vermeijden et al. [77]	24 (49)	23 (68)													0 (0)	0 (0)	0 (0)	0 (0)
FU ≥ 5 years																		
Hopper et al. [31]																		

	DIS	ACLR	DIS			ACLR			DIS			ACLR			DIS	ACLR	DIS	ACLR
Dynamic intraligamentary stabilisation																		
FU ≥ 2 years																		
Kayaalp et al. [38]	2 (13)	6 (20)																
Bieri et al. [4]	28 (53)	0 (0)	2			24			26			8						
FU ≥ 5 years																		
Hoogeslag et al. [27]	0 (0)	0 (0)	0			0												
Glasbrenner et al. [22]	12 (35)	6 (20)																

	BEAR	ACLR	BEAR			ACLR			BEAR			ACLR			BEAR	ACLR	BEAR	ACLR
Bridge‐enhanced ACL restoration																		
FU ≥ 2 years																		
Murray et al. [45]	23 (35)	0 (0)	8			4			15			15			0 (0)	1 (10)		
Murray et al. [47]	5 (50)	0 (0)	2	0	1	3	2	1	3	2	1	5	1	4				

*Note*: Data presented as *n* (%), if not indicated otherwise.

Abbreviations: ACL, anterior cruciate ligament; ACLPR, ACL primary repair; ACLR, ACL reconstruction; ALL, anterolateral ligament; BEAR, bridge‐enhanced ACL restoration; DIS, dynamic intraligamentary stabilisation; FU, follow‐up.; lat, lateral; MCL, medial collateral ligament; med, medial.

### ACL revision rates

#### ACLPR

**Figure 3:**
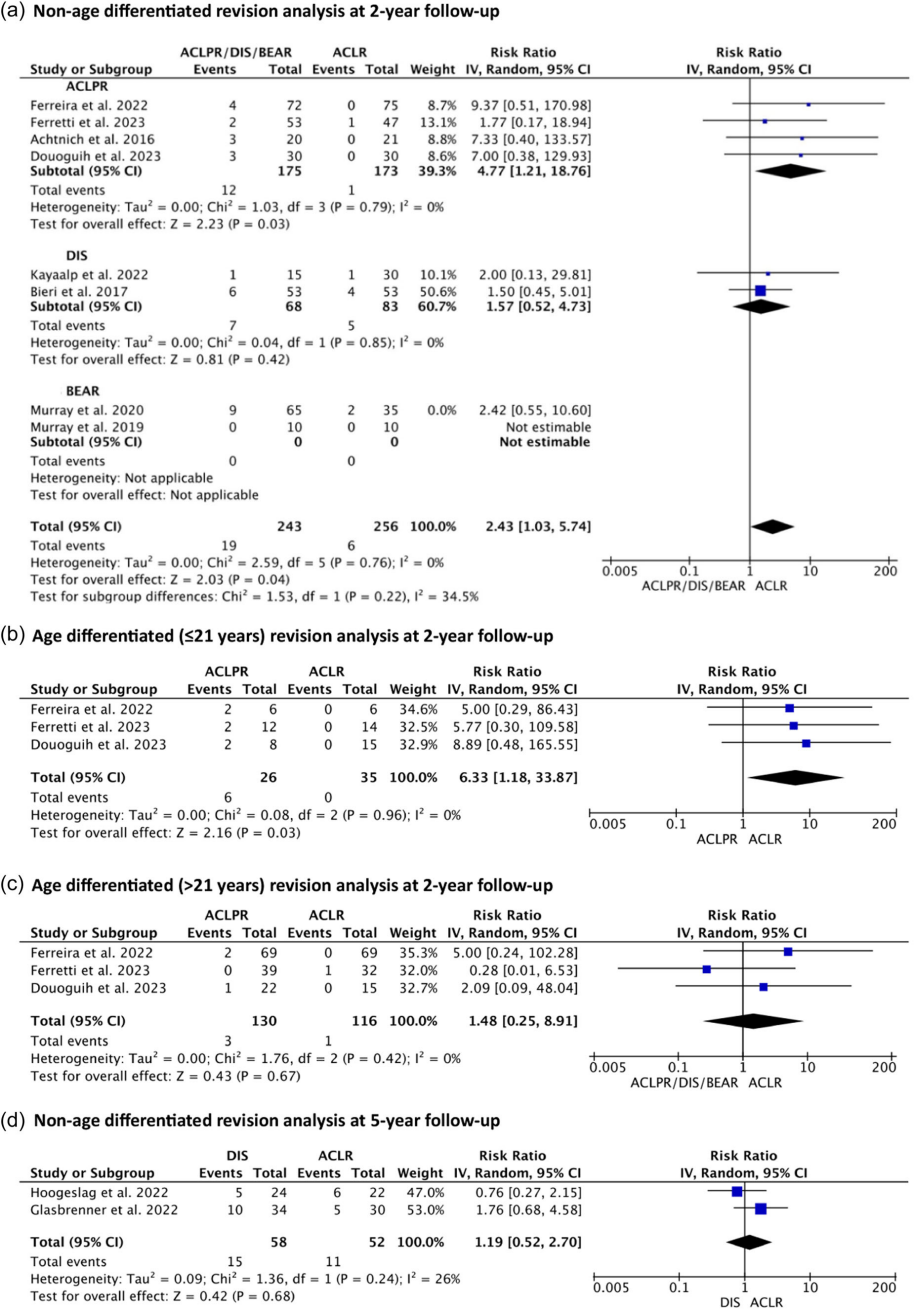
Relative risk random‐effects meta‐analysis: (a) ‘nonage‐differentiated anterior cruciate ligament (ACL) revision analysis for ACL primary repair (ACLPR), dynamic intraligamentary stabilisation (DIS) and bridge‐enhanced ACL restoration (BEAR) compared to ACL reconstruction (ACLR) at 2‐year follow‐up’, (b) ‘age differentiated (≤21 years) ACL revision analysis for ACLPR compared to ACLR at 2‐year follow‐up’, (c) ‘age differentiated (>21 years) ACL revision analysis for ACLPR compared to ACLR at 2‐year follow‐up’, (d) ‘nonage‐differentiated ACL revision analysis for DIS compared to ACLR at 5‐year follow‐up’.

Relative risk random‐effects meta‐analysis comparing ACLPR and ACLR revealed an increased risk for ACL revision for ACLPR at 2‐year follow‐up (*p* = 0.03) (Figure 3a). Most notably, the age‐stratified analysis revealed a markedly lower revision rate after ACLPR in adults (age >21) than skeletally mature patients ≤21 years of age: ACL revision risk for patients ≤21 years was significantly increased (*p* = 0.03) and patients >21 years showed no significant difference as compared to ACLR (n.s.) (Figure 3b,c).

#### DIS

Nonage‐stratified ACL revision risk for DIS, in the relative risk random‐effects meta‐analysis, demonstrated no significant differences as compared to ACLR at 2‐year (n.s.) (Figure 3a) and 5‐year follow‐up (n.s.) (Figure 3d). Age‐differentiated revision risk, presented in a tabulated fashion (Table 5), showed comparable results to ACLPR with increased failure rates for skeletally mature patients ≤21 years of age and low and comparable rates for adults (age >21) when compared to ACLR.

**Table 5:** Adverse events: IL ACL revision rates and CL ACL injury rates.

	ACL revision, all age			ACL revision, >21 years			ACL revision, ≤21 years			Failure time, months		Traumatic failure		CL ACL tear		
References	ACLPR	ACLR	Sign.	ACLPR	ACLR	Sign.	ACLPR	ACLR	Sign.	ACLPR	ACLR	ACLPR	ACLR	ACLPR	ACLR	Sign.
ACL primary repair																
FU ≥ 2 years																
Ferreira et al. [17]	4 (5)	0 (0)	**0.045**	2 (3)	0 (0)	0.157	2 (33)	0 (0)		13.5 ± 4.8		4 (100)		1 (1)	1 (1)	
Ferretti et al. [18]	2 (4)	1 (2)	0.63	0 (0)	1 (3)		2 (17)	0 (0)		28 ± 2.8	18	2 (100)	1 (100)	1 (2)	0 (0)	
Achtnich et al. [1]	3 (15)	0 (0)	**0.001**							20.0 ± 15.5		1 (33)				
Douoguih et al. [11]	3 (10)	0 (0)	0.24	1 (5)	0 (0)		2 (25)	0 (0)		13.3 ± 2.6		3 (100)		0 (0)	0 (0)	
Vermeijden et al. [77]^a^																
FU ≥ 5 years																
Hopper et al. [31]	22 (16)	32 (12)	0.194	11 (12)	21 (6)		3 (25)	11 (15)		26.6 ± 20.4	22.0 ± 15.5					

	DIS	ACLR	Sign.	DIS	ACLR	Sign.	DIS	ACLR	Sign.	DIS	ACLR	DIS	ACLR	DIS	ACLR	Sign.
Dynamic intraligamentary stabilisation																
FU ≥ 2 y																
Kayaalp et al. [38]	1 (7)	1 (3)		0 (0)	0 (0)		1 (20)	1 (8)		24.0 ± 0.0	36.0 ± 0.0	1 (100)	1 (100)			
Bieri et al. [4]	6 (11)	4 (8)	0.47									5 (83)	4 (100)			
FU ≥ 5 years																
Hoogeslag et al. [27]	5 (21)	6 (25)	0.731	1 (9)	1 (5)		4 (31)	5 (50)		29.0 ± 11.6	23.0 ± 13.1			3 (13)	0 (0)	
Glasbrenner et al. [22]	10 (29)	5 (17)	0.118							27.0 ± 6.8	29.0 ± 7.3					

	BEAR	ACLR	Sign.	BEAR	ACLR	Sign.	BEAR	ACLR	Sign.	BEAR	ACLR	BEAR	ACLR	BEAR	ACLR	Sign.
Bridge‐enhanced ACL restoration																
FU ≥ 2 years																
Murray et al. [45]	9 (14)	2 (6)	0.32													
Murray et al. [47]	0 (0)	0 (0)	NS													

*Note*: Data presented as *n* (%), if not indicated otherwise. Bold *p*‐values indicate significant differences.

Abbreviations: ACL, anterior cruciate ligament; ACLPR, ACL primary repair; ACLR, ACL reconstruction; BEAR, bridge‐enhanced ACL restoration; CL, contra‐lateral leg; DIS, dynamic intraligamentary stabilisation; FU, follow‐up; IL, ipsi‐lateral leg.

^a^ Study did not report on failure rates.

#### BEAR

Studies evaluating studies using the BEAR implant could not be pooled using relative risk random‐effects meta‐analysis, given that only two studies [45, 47] were available of which one study [47] was stated to be ‘not estimable’ as zero incidences were reported in both groups (Figure 3a). The revision risk for the one estimable study [45] stated an increased risk for BEAR as compared to ACLR at 2 years. Furthermore, there were no data available to further stratify this technique's outcomes by age.

### Concomitant Injuries, reoperation and removal of hardware (ROH)

**Figure 4:**
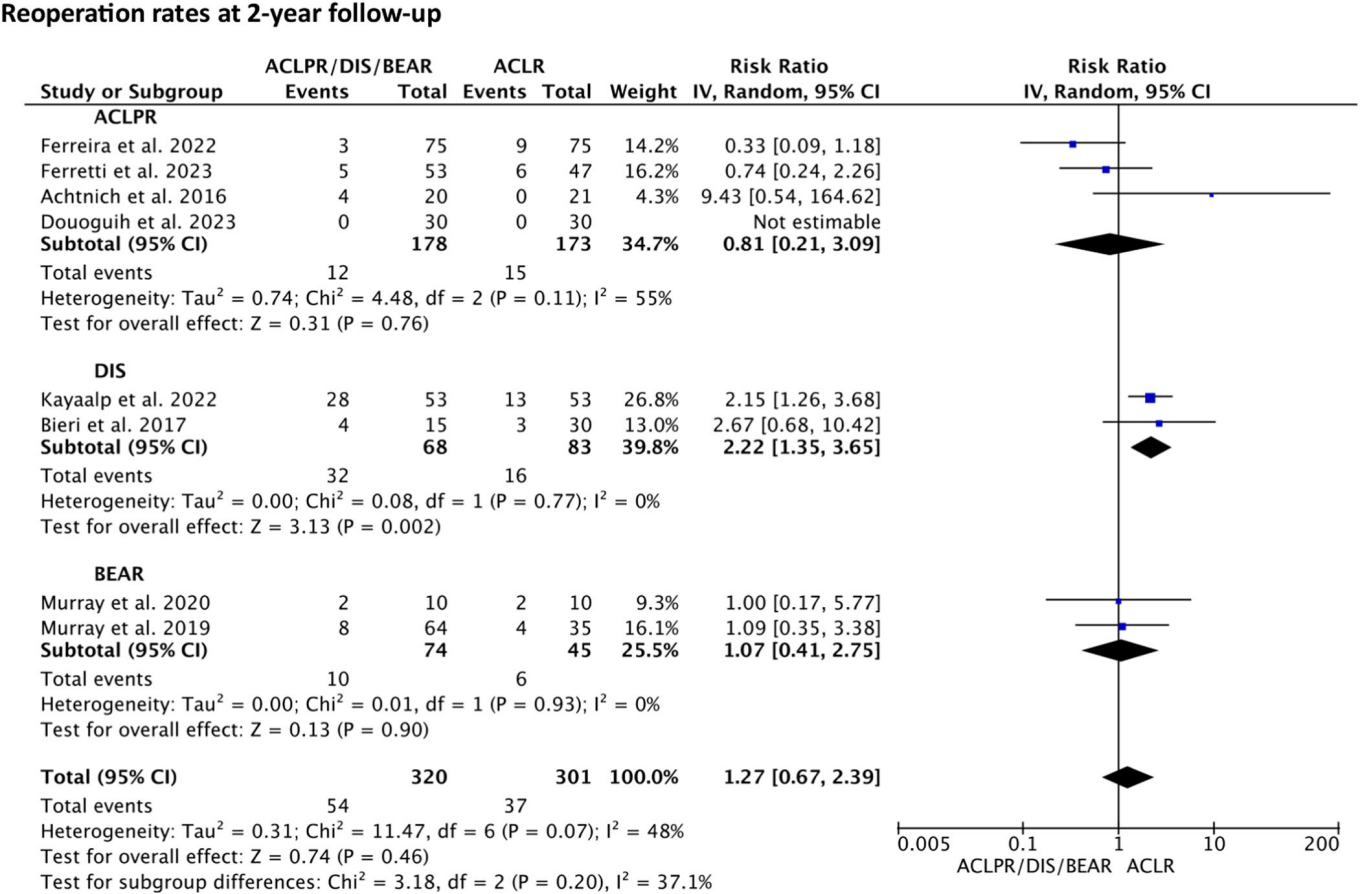
Relative risk random‐effects meta‐analysis: reoperation rates for anterior cruciate ligament primary repair (ACLPR), dynamic intraligamentary stabilisation (DIS) and bridge‐enhanced ACL restoration (BEAR) compared to ACLR at 2‐year follow‐up.

Reoperation rates (subsequent need for surgery on ipsilateral knee other than ACL revision) for DIS compared to ACLR were shown to be significantly increased (*p* = 0.002) [4, 22, 27, 38], whereas no significant difference and lower to equivalent rates were presented for ACLPR (n.s.) and BEAR (n.s.) (Figure 4). An extensive overview of all reoperations is provided in Table 6.

**Table 6:** IL reoperation rates.

	IL reoperations																			
	TOTAL			ROH		Meniscus			Cyclops syndrome		Chondroplasty		Arthrolysis		Manipulation		Lavage/Infect		Other	
References	ACLPR	ACLR	Sign.	ACLPR	ACLR	Sign.	ACLPR	ACLR	ACLPR	ACLR	ACLPR	ACLR	ACLPR	ACLR	ACLPR	ACLR	ACLPR	ACLR	ACLPR	ACLR
ACL primary repair																				
FU ≥ 2 years																				
Ferreira et al. [17]	3 (4)	9 (12)	0.183	3 (100)	0 (0)		0 (0)	1 (11)	0 (0)	8 (89)	0 (0)	0 (0)	0 (0)	0 (0)	0 (0)	0 (0)	0 (0)	0 (0)	0 (0)	0 (0)
Ferretti et al. [18]	5 (9)	6 (13)	0.59	2 (40)	0 (0)	0.50	2 (40)	1 (17)	0 (0)	0 (0)	0 (0)	0 (0)	0 (0)	1 (17)	1 (20)	1 (17)	0 (0)	3 (50)	0 (0)	0 (0)
Achtnich et al. [1]	4 (20)	0 (0)	**0.001**																	
Douoguih et al. [11]	0 (0)	0 (0)		0 (0)	0 (0)		0 (0)	0 (0)	0 (0)	0 (0)	0 (0)	0 (0)	0 (0)	0 (0)	0 (0)	0 (0)	0 (0)	0 (0)	0 (0)	0 (0)
Vermeijden et al. [77]^a^																				
FU ≥ 5 years																				
Hopper et al. [31]	5 (4)	12 (4)		0 (0)	0 (0)		5 (100)	5 (42)	0 (0)	0 (0)	0 (0)	2 (17)	0 (0)	0 (0)	0 (0)	1 (8)	0 (0)	3 (25)	0 (0)	1 (8)

	DIS	ACLR	Sign.	DIS	ACLR	Sign.	DIS	ACLR	DIS	ACLR	DIS	ACLR	DIS	ACLR	DIS	ACLR	DIS	ACLR	DIS	ACLR
Dynamic intraligamentary stabilisation																				
FU ≥ 2 years																				
Kayaalp et al. [38]	4 (27)	3 (10)		3 (75)	0 (0)		0 (0)	0 (0)	1 (25)	3 (100)	0 (0)	0 (0)	0 (0)	0 (0)	0 (0)	0 (0)	0 (0)	0 (0)	0 (0)	0 (0)
Bieri et al. [4]	28 (53)	13 (25)	0.67	19 (67)	3 (23)	**<0.001**	4 (14)	4 (31)	0 (0)	0 (0)	0 (0)	0 (0)	3 (11)	2 (15)	0 (0)	0 (0)	5 (18)	7 (54)	0 (0)	0 (0)
FU ≥ 5 years																				
Hoogeslag et al. [27]	9 (38)	4 (20)	0.205	3 (33)	0 (0)		1 (11)	1 (25)	3 (33)	3 (75)	0 (0)	0 (0)	0 (0)	0 (0)	0 (0)	0 (0)	2 (22)	0 (0)	3 (33)	0 (0)
Glasbrenner et al. [22]	4 (12)	4 (13)	0.4264	2 (50)	1 (25)		1 (25)	2 (50)	0 (0)	0 (0)	0 (0)	0 (0)	1 (25)	1 (25)	0 (0)	0 (0)	0 (0)	0 (0)	0 (0)	0 (0)

	BEAR	ACLR	Sign.	BEAR	ACLR	Sign.	BEAR	ACLR	BEAR	ACLR	BEAR	ACLR	BEAR	ACLR	BEAR	ACLR	BEAR	ACLR	BEAR	ACLR
Bridge‐enhanced ACL restoration																				
FU ≥ 2 years																				
Murray et al. [45]	8 (13)	4 (11)		1 (12)	0 (0)	≥0.99	7 (88)	0 (0)	0 (0)	0 (0)	0 (0)	0 (0)	0 (0)	0 (0)	0 (0)	0 (0)	0 (0)	0 (0)	0 (0)	0 (0)
Murray et al. [47]	2 (20)	2 (20)		1 (50)	0 (0)		1 (50)	1 (50)	0 (0)	1 (50)	0 (0)	0 (0)	0 (0)	0 (0)	0 (0)	0 (0)	0 (0)	0 (0)	0 (0)	0 (0)

*Note*: Data presented as *n* (%), if not indicated otherwise. Bold *p*‐values indicate significant differences.

Abbreviations: ACL, anterior cruciate ligament; ACLPR, ACL primary repair; ACLR, ACL reconstruction; BEAR, bridge‐enhanced ACL restoration; DIS, dynamic intraligamentary stabilisation; FU, follow‐up; IL, ipsi‐lateral leg.

^a^ Study did not report on reoperation rates.

### PROMs and clinical outcomes

Equivalent IKDC results were presented for ACLPR and BEAR at 2 years and DIS at 5 years. Significantly superior FJS results were shown for patients undergoing ACLPR at 2‐year follow‐up (*p* < 0.0001) and a significantly lower reduction in Tegner activity levels at 2 years was further shown for ACLPR when compared to ACLR (*p* < 0.00001). An extensive overview of all PROMs reported is provided in Figure 5 and Table 7; clinical outcomes (instrumented ATT SSD) are presented in Table 8.

Figure 5Random‐effects meta‐analysis of mean differences: (a) International Knee Documentation Committee (IKDC) 2‐year follow‐up, (b) IKDC 5‐year follow‐up, (c) Lysholm 2‐year follow‐up, (d) Forgotten Joint Score‐12 (FJS 2)‐year follow‐up, (e) Knee injury and Osteoarthritis Outcome Score (KOOS) 2‐year follow‐up, (f) Tegner 2‐year follow‐up, (g) Tegner 5‐year follow‐up.

**Figure:**
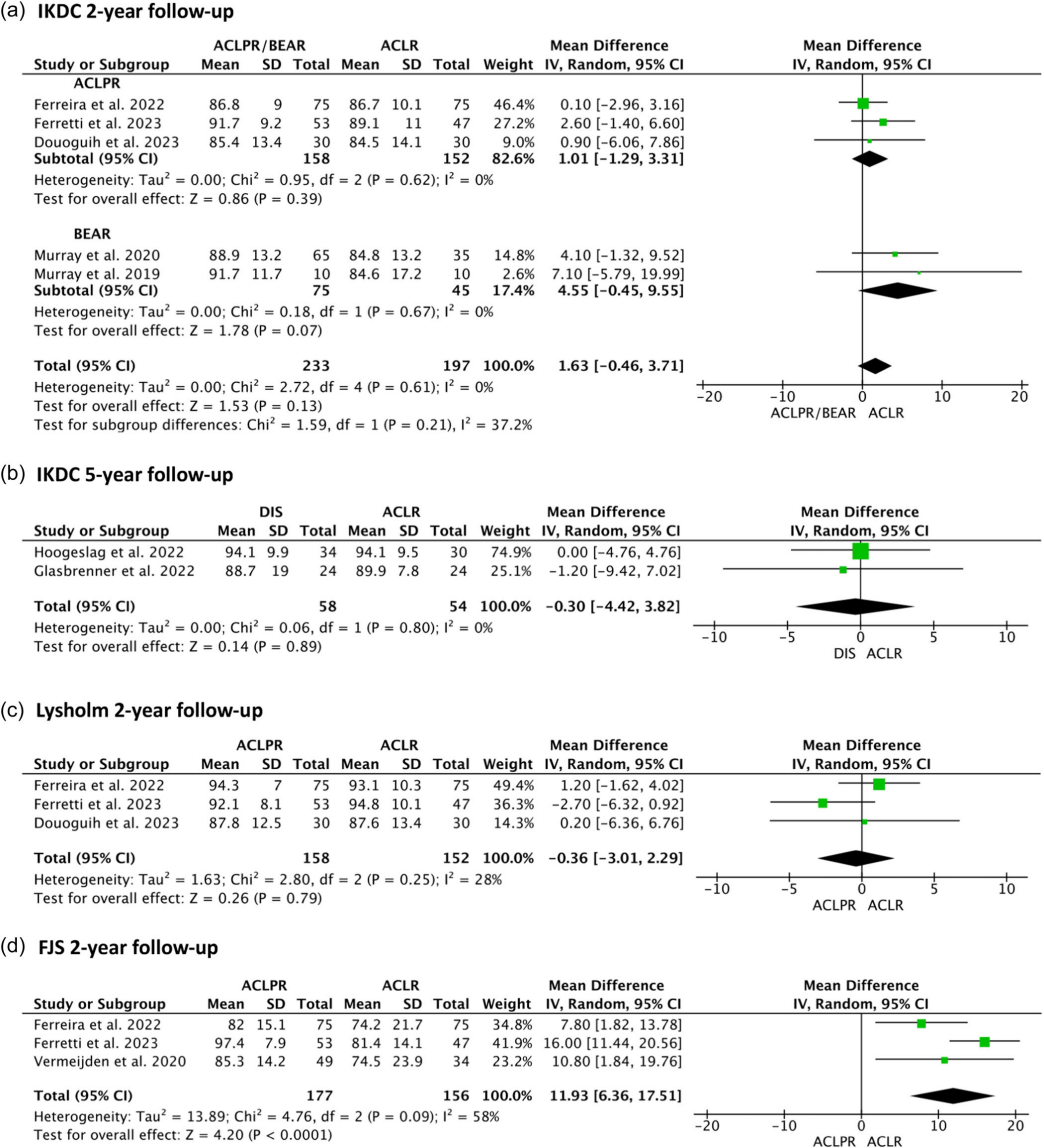

**Figure:**
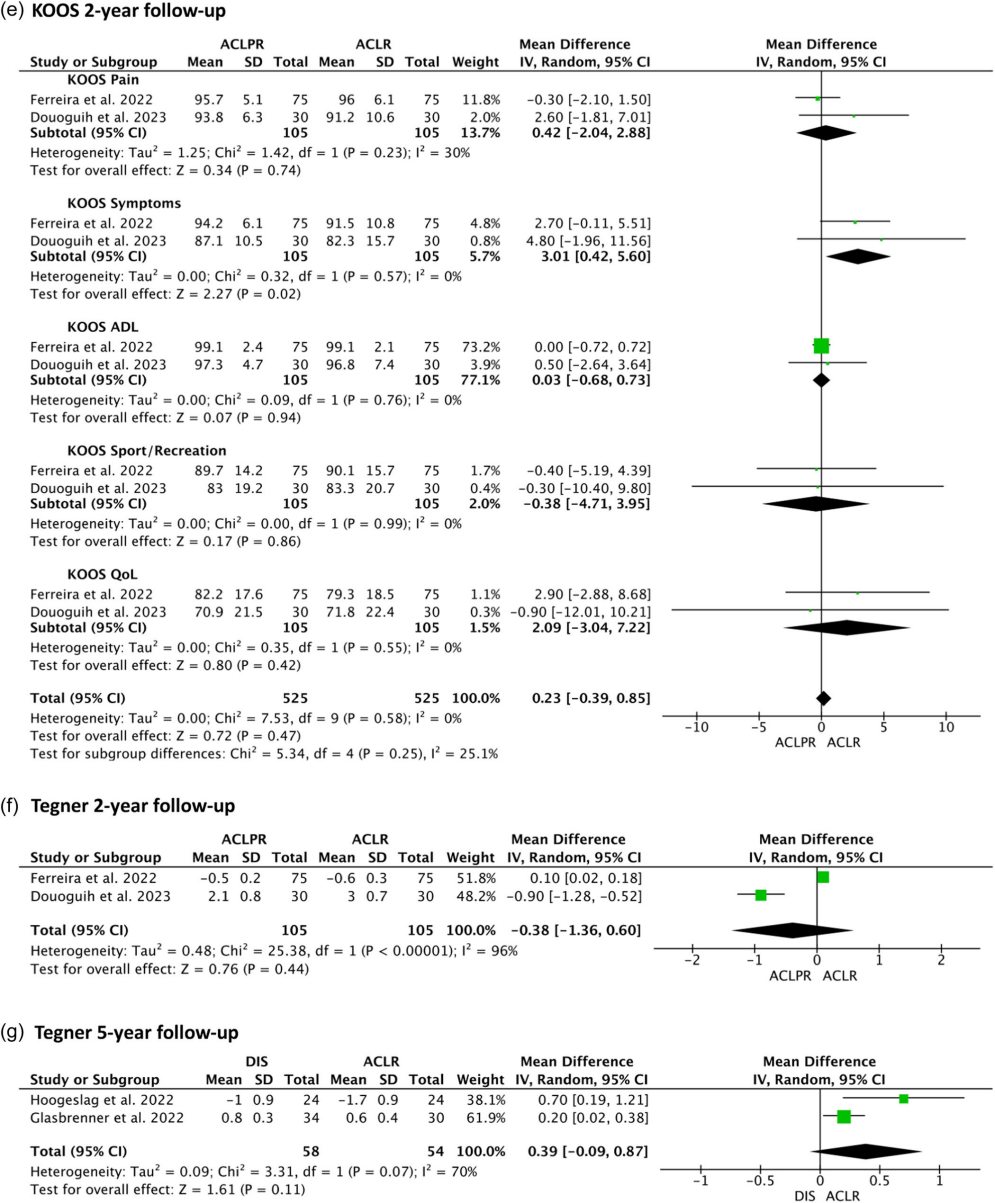

**Table 7:** Patient‐reported outcome measurements.

References	IKDC			Lysholm			FJS			Tegner difference		
	ACLPR	ACLR	Sign.	ACLPR	ACLR	Sign.	ACLPR	ACLR	Sign.	ACLPR	ACLR	Sign.
ACL primary repair												
FU ≥ 2 years												
Ferreira et al. [17]	86.8 ± 9.0	86.7 ± 10.1	>0.500	94.3 ± 7.0	93.1 ± 10.3	0.456	82.0 ± 15.1	74.2 ± 21.7	**0.017**	−0.5 ± 0.2	−0.6 ± 0.3	0.571
Ferretti et al. [18]	91.7 ± 9.2	89.1 ± 11.0	**<0.001**	92.1 ± 8.1	94.8 ± 10.1	0.22	97.4 ± 7.9	81.4 ± 14.1	**0.04**	0^§^	0^§^	0.838
Achtnich et al. [1]												
Douoguih et al. [11]	85.4 ± 13.4	84.5 ± 14.1	0.70	87.8 ± 12.5	87.6 ± 13.4	0.91				2.1 ± 0.8	3.0 ± 0.7	n.s.
Vermeijden et al. [77]							85.3 ± 14.2	74.5 ± 23.9	**0.022**			
FU ≥ 5 years												
Hopper et al. [31]												
	**DIS**	**ACLR**	**Sign.**	**DIS**	**ACLR**	**Sign.**	**DIS**	**ACLR**	**Sign.**	**DIS**	**ACLR**	**Sign.**
Dynamic intraligamentary stabilization												
FU ≥ 2 years												
Kayaalp et al. [38]	95.4 ± 2.8	94.6 ± 3.7	NS	96.3 ± 2.6	95.1 ± 3.0	NS				−0.7 ± 1.5	0.0 ± 0.8	n.s.
Bieri et al. [4]												
FU ≥ 5 years												
Hoogeslag et al. [27]	88.7 ± 19.0	94.1 ± 9.5								−1.0 ± 0.9	−1.7 ± 0.9	
Glasbrenner et al. [22]	94.1 ± 9.9	89.9 ± 7.8	**0.047**	97.0 ± 5.4	94.5 ± 5.5	0.322				0.8 ± 0.3	0.6 ± 0.4	
	**BEAR**	**ACLR**	**Sign.**	**BEAR**	**ACLR**	**Sign.**	**BEAR**	**ACLR**	**Sign.**	**BEAR**	**ACLR**	**Sign.**
Bridge enhanced ACL restoration												
FU ≥ 2 years												
Murray et al. [45]	88.9 ± 13.2	84.8 ± 13.2	0.15									
Murray et al. [47]	91.7 ± 11.7	84.6 ± 17.2	>0.05									

References	KOOS pain			KOOS symptoms			KOOS ADL			KOOS sport/recr.			KOOS QoL		
	ACLPR	ACLR	Sign.	ACLPR	ACLR	Sign.	ACLPR	ACLR	Sign.	ACLPR	ACLR	Sign.	ACLPR	ACLR	Sign.
ACL primary repair															
FU ≥ 2 years															
Ferreira et al. [17]	95.7 ± 5.1	96.0 ± 6.1	0.736	94.2 ± 6.1	91.5 ± 10.8	0.119	99.1 ± 2.4	99.1 ± 2.1	0.580	89.7 ± 14.2	*90.1* ± *15.7*	0.637	82.2 ± 17.6	79.3 ± 18.5	0.355
Ferretti et al. [18]	90.4	84.2		92.0	89.8		93.3	90.4		87.7	79.6		85.4	75	
Achtnich et al. [1]															
Douoguih et al. [11]	93.8 ± 6.3	91.2 ± 10.6	0.28	87.1 ± 10.5	82.3 ± 15.7	0.31	97.3 ± 4.7	96.8 ± 7.4	0.87	83.0 ± 19.2	83.3 ± 20.7	0.95	70.9 ± 21.5	71.8 ± 22.4	0.89
Vermeijden et al. [77]															
FU ≥ 5 years															
Hopper et al. [31]															
	**DIS**	**ACLR**	**Sign.**	**DIS**	**ACLR**	**Sign.**	**DIS**	**ACLR**	**Sign.**	**DIS**	**ACLR**	**Sign.**	**DIS**	**ACLR**	**Sign.**
Dynamic intraligamentary stabilization															
FU ≥ 2 years															
Kayaalp et al. [38]															
Bieri et al. [4]															
FU ≥ 5 years															
Hoogeslag et al. [27]	98.6 ± 1.5	97.2 ± 1.4	0.172	91.6 ± 2.7	95.5 ± 2.7	0.722	99.3 ± 0.7	100.0 ± 0.0	0.279	88.1 ± 19.2	96.6 ± 3.4	0.138	75.0 ± 37.1	86.3 ± 22.0	0.125
Glasbrenner et al. [22]															
	**BEAR**	**ACLR**	**Sign.**	**BEAR**	**ACLR**	**Sign.**	**BEAR**	**ACLR**	**Sign.**	**BEAR**	**ACLR**	**Sign.**	**BEAR**	**ACLR**	**Sign.**
Bridge enhanced ACL restoration															
FU ≥ 2 years															
Murray et al. [45]															
Murray et al. [47]	94.8 ± 11.7	90.5 ± 13.5		93.1 ± 9.4	85.2 ± 15.8		97.7 ± 5.8	98.3 ± 2.5		91.7 ± 14.4	85.7 ± 16.9		84.0 ± 15.7	70.5 ± 22.2	

*Note*: Data presented as pooled mean ± SD, if not indicated otherwise: ^§^median; underlined mean ± SD indicates insufficient score according to PASS thresholds; bold *p* values indicate significant differences.

Abbreviations: ACL, anterior cruciate ligament; ACLPR, ACL primary repair; ACLR, ACL reconstruction; BEAR, bridge‐enhanced ACL restoration; DIS, dynamic intraligamentary stabilisation; FJS, Forgotten Joint Score; FU, follow‐up; IKDC, International Knee Documentation Committee; KOOS, Knee injury and Osteoarthritis Outcome Score; KOOS ADL, KOOS activities of daily living; KOOS QoL, KOOS quality of life; KOOS Sport/Recr., KOOS Sport/Recreation; NS, not significant.

**Table 8:** Clinical outcomes.

	Instrumented ATT SSD			Measurement tool
References	ACLPR	ACLR	Sign.	
ACL primary repair				
FU ≥ 2 years				
Ferreira et al. [17]	1.1 ± 1.4	0.6 ± 1.0	**<0.0001**	Rolimeter
Ferretti et al. [18]	1.7 ± 0.3	1.9 ± 0.8	**<0.0001**	KT‐1000
Achtnich et al. [1]	2.0 ± 1.7	1.2 ± 0.7	0.269	KT‐1000
Douoguih et al. [11]	0.3 ± 1.0,^§^ 0.4 ± 0.9^#^	−0.2 ± 0.9,^§^ −0.3 ± 1.0^#^	0.084,^§^ 0.034^#^	KT‐1000
Vermeijden et al. [77]^a^				
FU ≥ 5 years				
Hopper et al. [31]^a^				

	DIS	ACLR	Sign.	
Dynamic intraligamentary stabilisation				
FU ≥ 2 years				
Kayaalp et al. [38]	1.9 ± 2.0	1.6 ± 1.2		
Bieri et al. [4]^a^				
FU ≥ 5 years				
Hoogeslag et al. [27]	1.0 ± 1.6	0.7 ± 0.8		Rolimeter
Glasbrenner et al. [22]	1.7 ± 1.6	1.4 ± 1.3	0.334	Rolimeter

	BEAR	ACLR	Sign.	
Bridge‐enhanced ACL restoration				
FU ≥ 2 years				
Murray et al. [45]	1.6 ± 3.2	1.8 ± 2.8	0.82	KT‐1000
Murray et al. [47]	1.9 ± 2.1	3.1 ± 2.7		KT‐1000

*Note*: Data presented as pooled mean ± SD; bold *p* values indicate significant differences.

Abbreviations: ACL, anterior cruciate ligament; ACLR, ACL reconstruction; ACLPR, ACL primary repair; ATT SSD, anterior‐tibial translational side‐to‐side difference; DIS, dynamic intraligamentary stabilisation; BEAR, bridge‐enhanced ACL restoration.

^a^ Study did not report on instrumented ATT SSD; study presented measurements taken with two different forces applied: ^§^15 and ^#^20 lbs of force.

### LOE and risk of bias assessment

**Figure 6:**
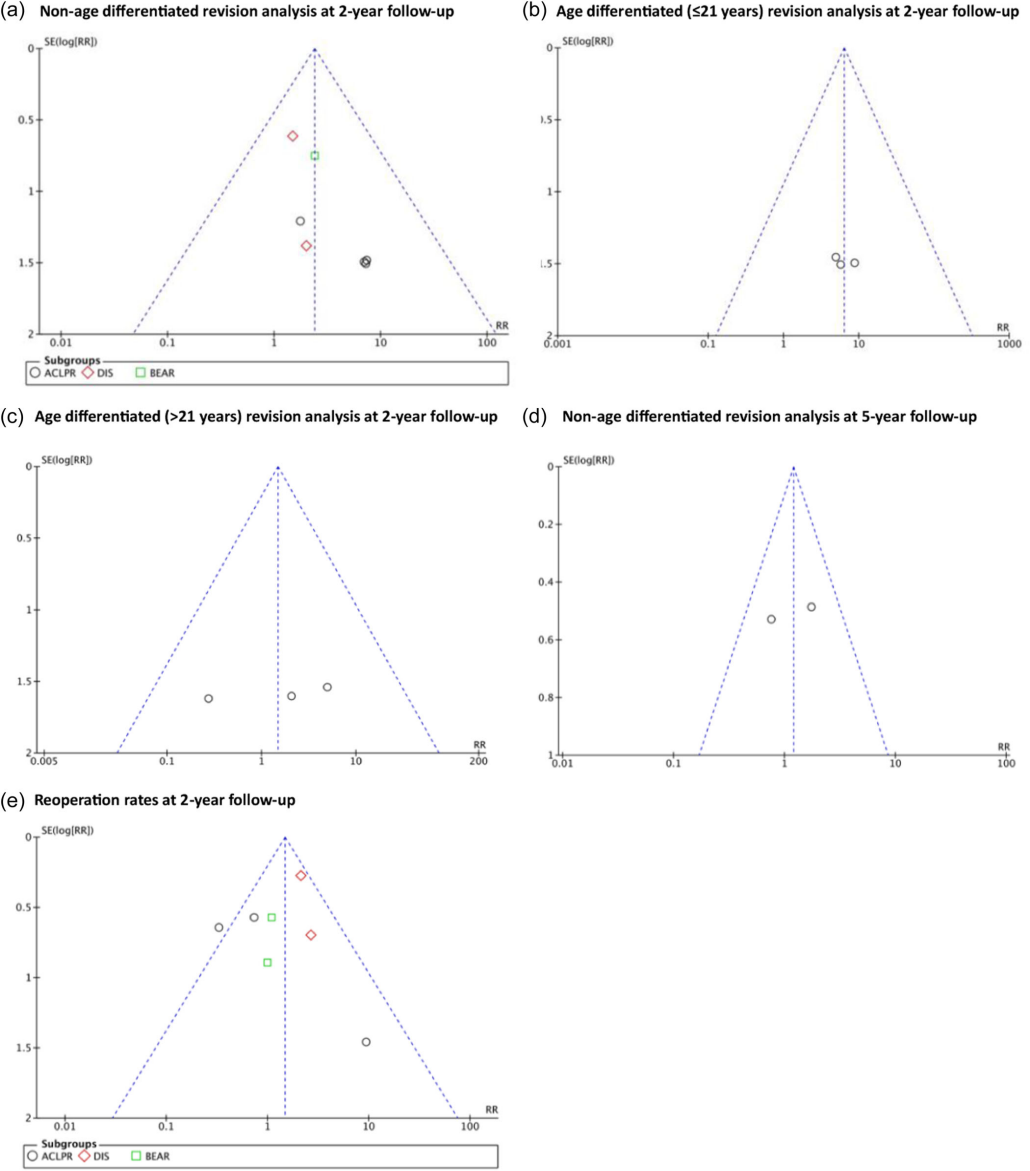
Publication bias ‘revision and reoperation rates’: Visual funnel plot analysis and significance levels calculated by Egger Test, (a) ‘nonage‐differentiated anterior cruciate ligament (ACL) revision analysis for ACL primary repair (ACLPR), dynamic intraligamentary stabilisation (DIS) and bridge‐enhanced ACL restoration (BEAR) compared to ACLR at 2‐year follow‐up’ (n.s.), (b) ‘age differentiated (skeletally mature patients ≤21 years of age) ACL revision analysis for ACLPR compared to ACL restoration (ACLR) at 2‐year follow‐up’ (n.s.), (c) ‘age‐differentiated (>21 years) ACL revision analysis for ACLPR compared to ACLR at 2‐year follow‐up’ (n.s.), (d) ‘nonage‐differentiated ACL revision analysis for DIS compared to ACLR at 5‐year follow‐up’, (e) ‘reoperation rates for ACLPR, DIS and BEAR compared to ACLR at 2‐year follow‐up’ showed potential visual and statistical evidence of publication bias (*p* = 0.017).

**Figure 7:**
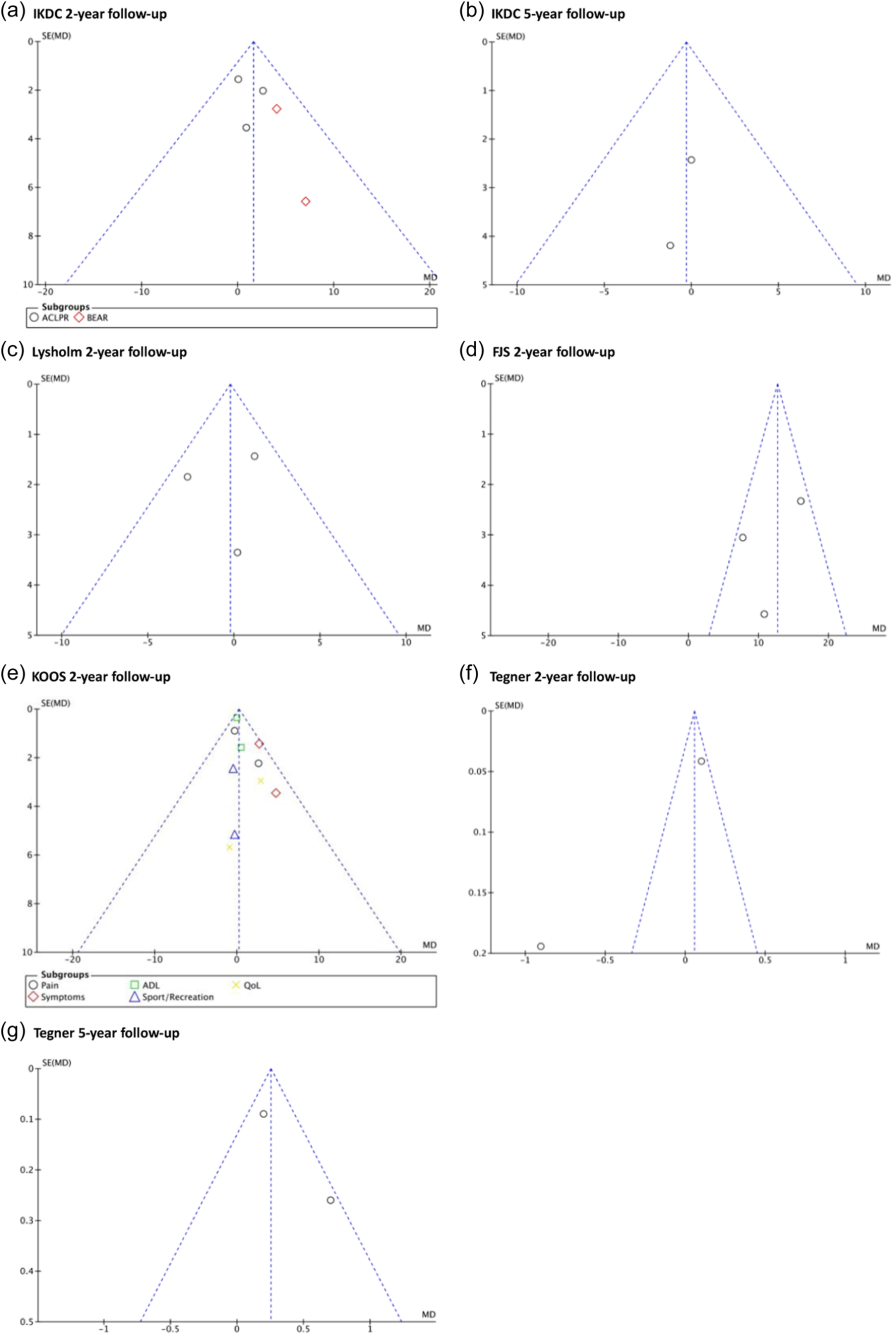
Publication bias ‘patient‐reported outcomes measurements (PROMs)’: Visual funnel plot analysis and significance levels calculated by Egger Test, (a) International Knee Documentation Committee (IKDC) 2‐year follow‐up (n.s.), (b) IKDC 5‐year follow‐up, (c) Lysholm 2‐year follow‐up (n.s.), (d) Forgotten Joint Score‐12 (FJS) 2‐year follow‐up (n.s.), (e) Knee injury and Osteoarthritis Outcome Score (KOOS) 2‐year follow‐up (n.s.), (f) Tegner 2‐year follow‐up, (g) Tegner 5‐year follow‐up.

The risk of bias of included RCTs, evaluated by the RoB 2, demonstrates overall ‘some concerns’ [69]. Nonrandomized trials presented an overall MINORS score of 20.3 ± 1.9 (18–24), suggesting a low risk of methodological bias [67]. The full and detailed risk of bias assessments are summarised in Tables 9 and 10. Additionally, funnel plot analysis and the Egger test (for outcomes with >2 studies) are presented in Figures 6 and 7.

**Table 9:** Methodological index for nonrandomized studies (MINORS).

References	1	2	3	4	5	6	7	8	9	10	11	12	Total (%)
ACL primary repair													
FU ≥ 2 years													
Ferreira et al. [17]	2	2	2	2	1	2	1	2	2	2	2	2	22 (92)
Ferretti et al. [18]	2	2	2	2	0	2	2	2	2	2	2	2	22 (92)
Achtnich et al. [1]	2	2	2	2	1	2	2	1	2	2	1	2	21 (88)
Douoguih et al. [11]	2	1	2	2	0	2	1	2	2	2	1	2	19 (79)
Vermeijden et al. [77]	2	2	2	2	0	2	1	1	2	2	1	2	19 (79)
FU ≥ 5 years													
Hopper et al. [31]	2	2	2	2	0	2	2	1	1	2	1	2	19 (79)
Dynamic intraligamentary stabilisation													
FU ≥ 2 years													
Kayaalp et al. [38]	2	0	2	2	1	2	1	2	2	2	2	2	20 (83)
Bieri et al. [4]	2	2	2	2	0	2	0	0	2	2	2	2	18 (75)
Bridge‐enhanced ACL restoration													
FU ≥ 2 years													
Murray et al. [47]	2	2	2	2	2	2	2	2	2	2	2	2	24 (100)

*Note*: MINORS Criteria: 0 (not reported), 1 (reported but inadequate) or 2 (reported and adequate). Minors Items 1–12: 1, a clearly stated aim; 2, inclusion of consecutive patients; 3, prospective collection of data; 4, endpoints appropriate to the aim of the study; 5, unbiased assessment of the study endpoint; 6, FU period appropriate to the aim of the study; 7, loss to follow‐up <5%; 8, prospective calculation of the study size; 9, an adequate control group; 10, contemporary groups; 11, baseline equivalence of groups; 12, adequate statistical analyses.

Abbreviations: ACL, anterior cruciate ligament; ACLPR, ACL primary repair; ACLR, ACL reconstruction; BEAR, bridge‐enhanced ACL restoration; DIS, dynamic intraligamentary stabilisation; FU, follow‐up; SA, suture augmentation.

**Table 10:** Cochrane risk‐of‐bias tool for randomized trials (RoB 2.0).

Authors	1	2	3	4	5	Overall
Dynamic intraligamentary stabilization						
FU ≥ 5 years						
Hoogeslag et al. [27]	Low	Low	Low	Some concerns	Low	Some concerns
Glasbrenner et al. [22]	Low	Some concerns	Low	Low	Low	Some concerns
Bridge‐enhanced ACL restoration						
FU ≥ 2 years						
Murray et al. [45]	Low	Some concerns	Low	Low	Low	Some concerns

*Note*: RoB 2.0 Tool: Risk‐of‐bias judgement (low/high/some concerns); 1, Domain 1 (randomization process); 2, Domain 2 (deviations from intended intervention); 3, Domain 3 (missing outcome data); 4, Domain 4 (outcome measurement); 5, Domain 5 (selection of the reported data).

Abbreviation: ACL, anterior cruciate ligament.

## DISCUSSION

The key findings of this systematic review indicate that, at a 2‐year follow‐up, ACLR demonstrated a lower nonage‐stratified revision risk compared to ACLPR, DIS and BEAR, while at 5‐year follow‐up, ACLR demonstrated a similar revision risk to DIS. However, an age‐stratified analysis revealed a significant difference in revision rates between skeletally mature patients ≤21 years of age and adults (age >21). ACLPR and DIS exhibited a significantly higher revision risk for patients aged ≤21 years compared to ACLR, whereas patients aged >21 years demonstrated a revision rate comparable to ACLR. This emphasises the necessity of considering age as a crucial risk factor in guiding treatment decisions for proximal ACL tears. Furthermore, reoperation rates for DIS were higher than those for ACLR, whereas BEAR and ACLPR showed no significant differences as to ACLR. Finally, ACLPR, DIS and BEAR all demonstrated equivalent IKDC scores when compared to ACLR. However, ACLPR exhibited significantly better FJS and KOOS Symptoms results, along with a lower reduction in Tegner activity levels at 2‐year follow‐up.

An age‐stratified revision risk analysis, using unpublished data from six studies [11, 17, 18, 27, 31, 38], stated significant difference between skeletally mature patients ≤21 years of age and adults (age >21), underscoring previous findings by Vermeijden et al. stating the age of 21 as an influential cutoff point for ACLPR revision risk [78]. This was further confirmed by Ferreira et al. in their ACLR versus ACLPR matched pair analysis, of a cohort of patients between the age of 16 and 64. Significantly higher nonage‐differentiated revision rates were demonstrated using ACLPR, however no significant differences when performing an age‐stratified subgroup analysis and only considering adults (>21 years) [17]. Regarding the BEAR, unfortunately, no data were available for age stratification in this meta‐analysis. However, prior BEAR studies align with the quantitative analysis findings for ACLPR and DIS in this study. BEAR revision rates in cohorts <16, 16–17 and 18–22 years of age, were shown to be 26.3%, 22.5% and 12.1%, respectively, compared to 0.0% in patients aged >22, as odds for revision were stated to decrease by one‐third for each 1‐year increase in age [63].

Nonetheless, it is important to note that an increased reinjury risk among younger patients is not unique to ACLPR, DIS, or BEAR, but also a topic of concern in ACLR. ACLR has shown two‐ to sixfold increased reinjury rates in younger patients, with studies using age cutoffs between 18 and 25 years [14, 30, 35, 56, 62, 68, 80, 81]. The Multicenter Orthopaedic Outcomes Network group emphasised this by concluding that the odds of ACLR reinjury decrease by 0.09 for every year of age increase [35]. However, authors have also hypothesised that age may only be a surrogate for increased risk‐taking and exposure to higher activity levels in young patient populations [63], referring to animal studies showing improved migration, proliferation and ACL healing in younger animals [41, 48]. Therefore, the correlation between ACL revision rates, age and activity level must be further investigated in future studies to improve patient selection.

To mitigate ACLR revision rates in young and high‐risk patients, combining ACLR with lateral stabilisation procedures has been demonstrated to reduce reinjury rates by over threefold [20, 21, 56, 61]. This has also been shown by two ACLPR studies included in this review, utilising ALL augmentation [28] and ALL repair [18]. Hopper et al. presented an ACL revision rate of 5.3% in a case series of 43 young active high‐risk patients (Marx acitivity scale >14) [28] and Ferretti et al. [18] presented no significant revision rate difference (n.s.) in a comparative study of 100 patients between ACLPR with ALL repair and ACLR with lateral extra‐articular tenodesis, at 3.8% and 2.1%, respectively. Therefore, the addition of an ALL augmentation or repair could be considered to improve success rates for modern‐day ACL repair, especially in young and active patients. However, more research is needed in this regard.

Considering technique‐specific reoperation rates, this review revealed significantly increased rates for DIS when compared to ACLR. In contrast, no significant difference and lower to equivalent rates were presented for ACLPR and BEAR. Notably, the majority of reoperation rates in DIS have been reported to be due to ROH, accounting for up to 75% of the performed reoperations [4, 22, 27, 38]. This can be related to the technique‐specific use of the Ligamys implant, a tibial fixation screw of 30 mm length and 10 mm diameter with an integrated spring system providing dynamic suture augmentation. This differentiates DIS [13] from ACLPR [1, 10, 23, 51, 59, 83] and BEAR [47], which both use smaller suture anchors to achieve ‘static’ suture augmentation fixation. The discrepancy in reoperation rates between ACLPR, DIS and BEAR highlights the importance of differentiating available repair techniques in future research, which has previously been neglected [49, 53, 65].

Finally, when reviewing PROMs between the respective repair techniques and ACLR, this study showed equivalent IKDC scores for all evaluated techniques, while ACLPR additionally demonstrated significantly better FJS and KOOS Symptoms results. This aligns with the findings of previous systematic reviews and meta‐analyses demonstrating good to excellent subjective outcomes [53, 73, 76, 82]. A major factor influencing patient satisfaction following surgery is pain [50]. Given the minimally invasive nature of modern‐day repair approaches compared to ACLR, with no need of graft harvesting or tunnel drilling, ACL repair minimises donor‐site morbidity and therefore pain levels [75, 77]. This has been shown in a cohort study comparing ACLPR and ACLR, demonstrating lower postoperative pain levels and significantly lower opiod intake by the ACLPR cohort, while at the same time stating higher recovery quality and earlier return of full ROM [75].

This study is subjected to several general limitations, as characteristic to systematic reviews and meta‐analyses, including publication and selection bias, which pose the risk of not capturing all relevant studies and the potential for data to be inaccurately or incompletely extracted. To mitigate these risks, this study adhered to standard analytical methodologies [7, 52]. Furthermore, and more specifically concerning the included studies, it is essential to acknowledge that, due to the low number of available high LEO studies and the necessity to exclude multiple LOE 4 and noncomparative studies, the quantitative analysis was restricted to a small number of LOE 1–3 studies and a high number of retrospective studies. To address this limitation, a detailed risk of bias analysis was performed, demonstrating no high concerns in any of the included studies. Additionally, ACLR techniques of the included studies are heterogenous, given that different graft types were included. To mitigate the potential impact of concomitant injuries, the authors sought additional information from authors of studies that did not sufficiently report on this factor by reaching out to them through email. The additionally gathered information has been incorporated into a comprehensive breakdown of the types of meniscus treatments and other concomitant injuries in Table 4. Also, differences in patient populations exist regarding age, sex, surgery delay and activity levels and only 30% out of the non‐RCT performed cohort matching. To consider and accommodate for heterogeneity among the evaluated studies, a random‐effects meta‐analysis was conducted to account for both within‐study and between‐study variability [7, 9]. However, given the potential heterogeneity in ACLR approaches and patient populations in general, caution should be excercised when comparing revision rates of the respective repair techniques in this meta‐analyis based solely on the presented relative revision RR. The variations in pooled relative ACLR revision RR, with only 0.05% in studies with ACLPR comparators but 6.0% in DIS cohorts, potentially impacted the ACLPR and DIS relative revision RR presented in this meta‐analysis.

Finally, it should be emphasised that it is crucial for future research to continue to define risk factors for ACL repair. This is essential to ensure that clinical practice remains evidence‐based and to prevent history from repeating itself. Therefore, the results of this review are of clinical relevance and should provide clinicians with extended knowledge regarding the risk factor age, helping to further refine the indication spectrum for ACLPR and to guide the treatment decision‐making for proximal ACL tears.

## CONCLUSIONS

ACLPR in skeletally mature patients ≤21 years of age is associated with up to a sixfold risk increase for ACL revision surgery compared to ACLR; however, adults (>21 years), present no significant difference. Based on the current data, age emerges as a crucial risk factor and should be considered when deciding for the appropriate treatment option in proximal ACL tears.

## AUTHOR CONTRIBUTIONS

**Sebastian Rilk**: Conceptualisation; data acquisition and analysis; manuscript writing. **Gabriel C. Goodhart**: Data acquisition; manuscript editing. **Jelle P. van der List**: Conceptualisation; manuscript editing. **Fidelius Von Rehlingen‐Prinz**: Data acquisition; manuscript editing. **Harmen D. Vermeijden**: Manuscript editing. **Robert O'Brien**: Manuscript editing. **Gregory S. DiFelice**: Conceptualization; manuscript editing.

## CONFLICT OF INTEREST STATEMENT

The authors declare no conflict of interest.

## ETHICS STATEMENT

The authors have nothing to report.

## Data Availability

All data supporting the findings of this study are available within the paper and its Supporting Information.
